# Immunological Cross-Reactivity and Preclinical Assessment of a Colombian Anticoral Antivenom against the Venoms of Three *Micrurus* Species

**DOI:** 10.3390/toxins16020104

**Published:** 2024-02-15

**Authors:** Ariadna Rodríguez-Vargas, Adrián Marcelo Franco-Vásquez, Miguel Triana-Cerón, Shaha Noor Alam-Rojas, Derly C. Escobar-Wilches, Gerardo Corzo, Fernando Lazcano-Pérez, Roberto Arreguín-Espinosa, Francisco Ruiz-Gómez

**Affiliations:** 1 Grupo de Investigación en Animales Ponzoñosos y sus Venenos, Instituto Nacional de Salud, Bogotá 111321, Colombia; mitriana@udca.edu.co (M.T.-C.); snalamr@unal.edu.co (S.N.A.-R.); fruiz@ins.gov.co (F.R.-G.); 2 Departamento de Química de Biomacromoléculas, Instituto de Química, Universidad Nacional Autónoma de México, Coyoacán, Mexico City 04510, Mexico; adrian.franco.vasquez@gmail.com (A.M.F.-V.); ferlaz@comunidad.unam.mx (F.L.-P.); arrespin@unam.mx (R.A.-E.); 3 Bacterial Molecular Genetics Laboratory, Research Department, Universidad El Bosque, Bogotá 110121, Colombia; 4 Facultad de Medicina, Fundación Universitaria Sanitas, Bogotá 110131, Colombia; dcescobarwi@unisanitas.edu.co; 5 Departamento de Medicina Molecular y Bioprocesos, Instituto de Biotecnología, Universidad Nacional Autónoma de México, Cuernavaca 62210, Mexico; gerardo.corzo@ibt.unam.mx

**Keywords:** snake venoms, antivenoms, *Micrurus* sp., immunorecognition, neutralization

## Abstract

Snakebite accident treatment requires the administration of antivenoms that provide efficacy and effectiveness against several snake venoms of the same genus or family. The low number of immunogenic components in venom mixtures that allow the production of antivenoms consequently gives them partial neutralization and a suboptimal pharmacological response. This study evaluates the immunorecognition and neutralizing efficacy of the polyvalent anticoral antivenom from the Instituto Nacional de Salud (INS) of Colombia against the heterologous endemic venoms of *Micrurus medemi*, and *M. sangilensis*, and *M. helleri* by assessing immunoreactivity through affinity chromatography, ELISA, Western blot, and neutralization capability. Immunorecognition towards the venoms of *M. medemi* and *M. sangilensis* showed values of 62% and 68% of the protein composition according to the immunoaffinity matrix, respectively. The analysis by Western blot depicted the highest recognition patterns for *M. medemi*, followed by *M. sangilensis*, and finally by *M. helleri*. These findings suggest that the venom compositions are closely related and exhibit similar recognition by the antivenom. According to enzyme immunoassays, *M. helleri* requires a higher amount of antivenom to achieve recognition than the others. Besides reinforcing the evaluation of INS antivenom capability, this work recommends the use of *M. helleri* in the production of Colombian antisera.

## 1. Introduction

The inoculation of venom after a snakebite accident introduces a variety of toxic molecules that injure tissues and cause several pathophysiological changes in the victim [1]. In a global panorama, it is estimated that there are more than 1,800,000 new cases per year, of which approximately 90,000 end in fatal events, with Asia, Africa, and Latin America being the most affected geographic regions [2]. In 2022, the number of cases in Colombia was 5573, with an incidence of 10.8 per 100,000 inhabitants, an accumulated lethality of 0.57%, and antivenom application in 85% of cases. Therefore, snakebite accidents are considered an event of interest in public health, in which the Orinoquía, Amazonía, Andina, and Pacific Coast regions stand out as the most affected [3].

Colombia harbors several species pertaining to the three large families of snakes that cause snakebite accidents: viperids, elapids, and colubrids [4]. Elapids on the American continent are represented by the genus *Micrurus*, which is widely distributed from the Southeastern United States to Northern Argentina [5]. Coral snakes are easily recognized by their characteristic color patterns, which are allocated as monads, dyads, triads, or even incomplete rings [6,7]. These snakes are predominantly nocturnal [8], with a diet that includes fish, other snakes, and small reptiles [9]. To date, approximately 31 species of *Micrurus* have been reported in Colombia [10], widely distributed throughout the country, predominantly in the Pacific and Amazonía regions [5]. Coral snakes cause approximately 1% of snakebite accidents per year [11], and, although their bites are less frequent than those of viperids, given their non-aggressive behavior [7], they are considered serious due to the neurotoxic components that affect molecular targets at the neuromuscular junction, leading to respiratory paralysis and even death if a patient is not treated adequately in time [12].

An existing pragmatic classification of the genus *Micrurus* allows its arrangement into groups according to color patterns. According to this classification system, *M. dumerilii* and *M. mipartitus* are included in the monad and bicolor groups in Colombia, respectively [5,13,14]. In order to increase the knowledge of the species comprised within the groups and to determine the similarities or differences within each one, the venoms of *M. medemi* and *M. sangilensis* were selected as examples of monadic patterns, whereas the venom of *M. helleri* was selected as an example of a triadic pattern.

*M. helleri* (LINNAEUS, 1758) is distributed on the eastern side of the Cordillera Oriental, Orinoquía, and Amazon ecoregions of Colombia [5]; *M. medemi* (ROZE, 1967) also inhabits the eastern side of the Cordillera Oriental, although it is only known to be found in the Meta Department, being a common coral snake in the vicinity of the urban and peri-urban areas of Villavicencio [15,16,17]; and *M. sangilensis* (NICÉFORO MARIA, 1942) is a coral snake restricted to elevations between 800 and 2000 meters above sea level of the Middle Magdalena River Basin, reported only in the Santander (type locality: municipality of San Gil), Boyacá, and northern Cundinamarca Departments [18].

Clinical management requires the administration of antivenoms of equine origin [19], which provide direct or cross-immunoreactivity against venoms from multiple species of snakes belonging to the same family [20]. The diversification of snake venoms from the *Micrurus* genus has been described in several reports, including evidence of the presence of isoforms and the relative abundances of the main components [13,21]. For instance, the most important components in *Micrurus* venoms are the three-finger toxins (3FTxs) and PLA_2_s, which are ubiquitous in all *Micrurus* species analyzed to date [22]. The abundance of these toxins depends on the species and may vary from 80% 3FTxs in species like *M. corallinus* and *M. tschudii* to ca. 20% in *M. dumerilii*. The same variation has been observed for PLA_2_s. The venoms also contain different proportions of metalloproteases, serine proteases, LAOOs, Kuntiz-type peptides, and C-type lectins, among others [23,24]. These differences in protein proportions and chemical variations could be the result of multicausal factors such as phylogeographic distribution, snake life stage, diet, and sexual dimorphism, among others [25,26,27,28]. The variability in the venom contents and the molecular characteristics of the proteins also affect their immunogenicity [29,30,31,32], causing partial or no neutralization by existing antivenoms, as well as suboptimal pharmacological responses in clinical management [33,34,35].

The assessment of antivenoms based on preclinical tests includes lethality neutralization as the gold standard; however, some complementary in vivo and in vitro methodologies, such as the neutralization of specific toxic activities, enzyme immunoassays, immunochemical trials, and antivenomics, complement antivenom evaluation in a robust way, allowing reproducibility, high sensitivity, low cost, and the implementation of alternative methodologies to the use of animals [36,37,38,39,40].

Multifactorial differences in venom composition and other factors, such as the size, sex, and age of the snake [26], determine a patient’s response to antivenoms. The anticoral antivenom from the Instituto Nacional de Salud (INS) of Colombia has been proven to neutralize the venom of most of the medically important *Micrurus* species, such as *Micrurus dumerilii*, *M. mipartitus*, *M. isozonus*, *M. surinamensis*, *M. medemi*, *M. helleri*, and *M. spixii* [41,42]. However, there are unresolved features regarding its scope for other existing *Micrurus* species. Therefore, it is necessary to complement its assessment with some preclinical studies. This study aimed to evaluate the immunorecognition and neutralization capacity of the INS coral snake antivenom against *M. medemi*, *M. sangilensis*, and populations of *M. helleri* venoms from Colombia, given the variability associated with interspecific and geographic distribution factors. Also, this study is complementary to a previous work that described the proteomic findings of the venoms of the same three species [23].

## 2. Results and Discussion

### 2.1. Preferential Recognition towards Complete Venoms of the Monad Group

**Figure 1:**
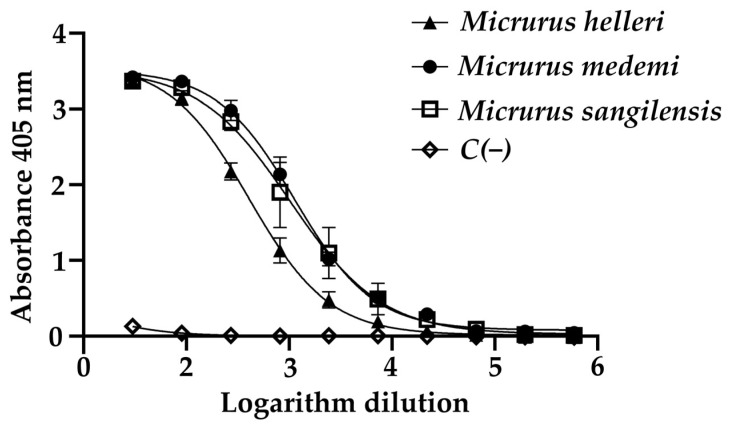
Cross-immunorecognition through ELISA assay of INS polyvalent anticoral antivenom against the crude venoms of *Micrurus helleri*, *M. medemi*, and *M. sangilensis*. IgG of non-immunized horses was used as negative control {C(−)}. *p*-value = 0.05. Each point represents the average of three measurements.

The median effective concentration (EC_50_) of the INS anticoral antivenom for the recognition of the venoms in the ELISA assay was determined by calculating the log_10_ of the antivenom dilutions (Figure 1). The *M. helleri* venom (406.9 ± 1.5) exhibited less recognition by the antivenom compared to *M. sangilensis* (1024 ± 1.8) and *M. medemi* (1136 ± 2.0); the latter two showed immunogenic similarity. The mechanism of cross-recognition has been described for the antivenom, manufactured by the Instituto Clodomiro Picado (ICP) of Costa Rica. This antivenom immunoreacts directly against *M. nigrocinctus* venom and exerts cross-immunoreactivity recognition of the venom of *M. clarkii* [22]. These characteristics indicate a high degree of conserved immunogenicity between both venoms. The same mechanism could explain the activity of the Colombian antivenom against the venoms of the monadic species *M. medemi* and *M. sangilensis* [5,6,7].

The differential immunoreactivity observed for antivenoms is related to the interspecific variations in the venoms, as reported by Rodríguez et al., 2023, in a proteomic approach study [23]. Larger proteins (with higher immunogenicity) will generate different immune response profiles to antivenom [43,44]. These interspecific differences have been reported for the Probiol antivenom [45], also produced in Colombia, which is capable of neutralizing the effect of the venom of *M. dumerilii* but not *M. mipartitus* venom [46]. An interesting fact is the neutralizing effect observed for the Australian polyvalent antielapidic antivenom, which is mostly used to treat *Oxyuranus scutellatus*, *Pseudechis australis*, and *Notechis scutatus* bites. Despite the fact that *Micrurus* snakes are endemic to the American continent, this antivenom shows marked effectiveness against most venoms from *Micrurus* species, except for *M. spixii* venom, due to important differences in its composition [47].

### 2.2. High-Molecular-Weight Components Are Better Immunogens

**Figure 2:**
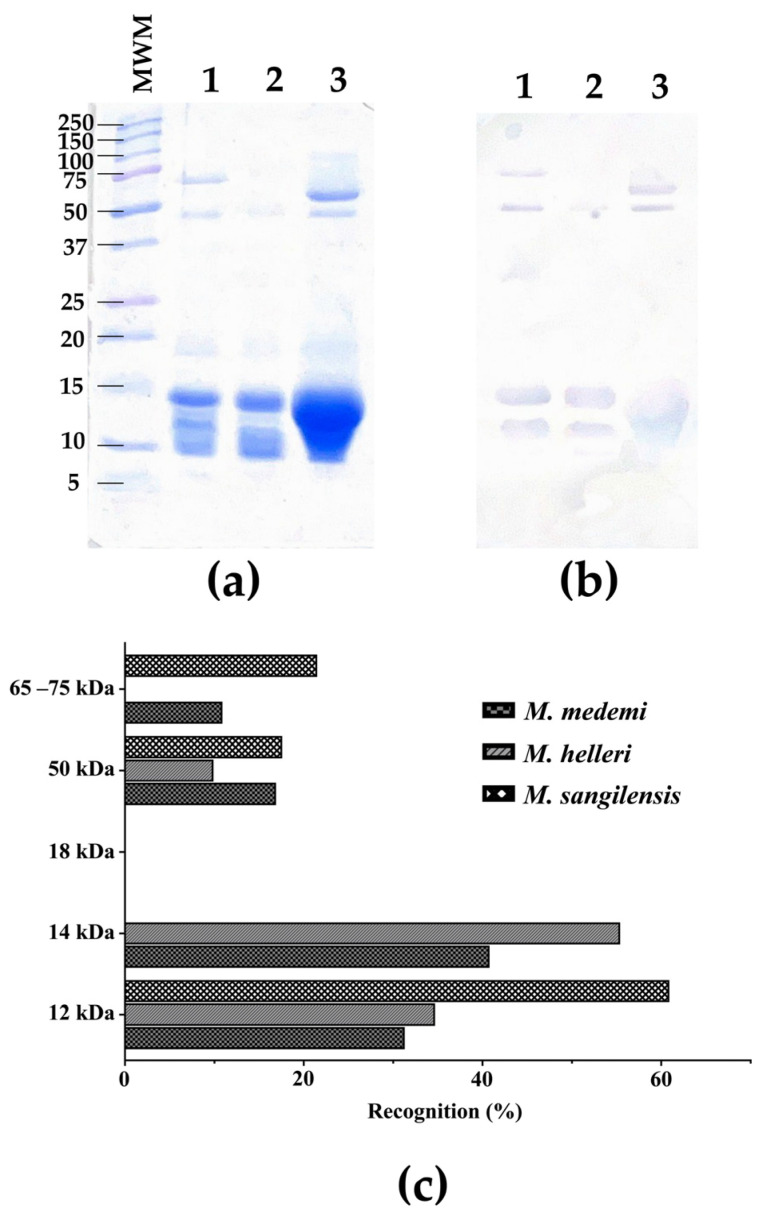
Electrophoretic and immunorecognition profiles. (**a**) SDS-PAGE, 15% of the three venoms under reducing conditions (MWM: Molecular Weight Marker); (**b**) Western blot of INS polyvalent anticoral antivenom against the venoms of three Colombian *Micrurus* snakes. *Micrurus medemi* (lane 1), *M. helleri* (lane 2), and *M. sangilensis* (lane 3). (**c**) Western blot densitometry analysis using GelAnalyzer software v. 23.1.1 (available at www.gelanalyzer.com by Istvan Lazar Jr., PhD and Istvan Lazar Sr., PhD, CSc).

In order to assess the effects of molecular weights and the immunoreactivity against some of the specific components of the three venoms tested, an SDS-PAGE along with a Western blot analysis were performed (Figure 2a,b). The results showed a degree of immunorecognition by the antivenom, exhibiting immunoaffinity towards certain bands. This analysis shows that the antivenom recognizes components of the three venoms tested in the 40–100 kDa and 12–15 kDa ranges. However, the bands in the 10 kDa and the 15–37 kDa range seem not to be recognized (Figure 2b).

The recognition and the neutralizing activity of an antivenom are also related to some characteristics of the immunogenic compounds in the venom, such as the molecular weight of proteins. Low-molecular-weight proteins (e.g., 3FTx) [48,49] show lower immunogenicity than proteins with a higher molecular weight (e.g., PLA_2_, LAAO) [43]. The bands corresponding to medium-molecular-weight proteins from the venoms of *M. medemi* and *M. sangilensis* were mostly recognized by the INS antivenom, specifically in the 45–75 kDa and 10–15 kDa ranges. In contrast, the high-molecular-weight components of the same venom were weakly recognized (Figure 2b). This was corroborated by a densitometry quantitative analysis (Figure 2c). The high-molecular-weight bands (>50 kDa) are related to proteases and LAAOs [50]. In a previous work [23], the high-molecular-weight fraction of *M. sangilensis* venom represents ca. 27%, a fraction that is recognized at approximately 38% by the antivenom. Likewise, this fraction represents approximately 27% of *M. helleri* venom, although it exhibited only 10% antivenom recognition. The medium-molecular-weight components (~14 kDa) comprise PLA_2_ and C-type lectins and represent a venom composition of ca. 40% for *M. helleri* and 50% for *M. medemi*, with 55% and 40% antivenom recognition, respectively. The *M. sangilensis* low-molecular-weight fraction (~12 kDa) did not exhibit antivenom recognition; however, its low-molecular-weight fraction (~10 kDa) showed 60% antivenom recognition. This fraction represents ca. 18% of the proteome and includes 3FTxs [23]. These results were also supported by the ELISA values (Figure 1), where the *M. helleri* venom showed less recognition than the *M. medemi* and *M. sangilensis* venoms.

### 2.3. Hydrophobicity and Large Molecular Size as Determinants of Recognition by the INS Antivenom

**Figure 3:**
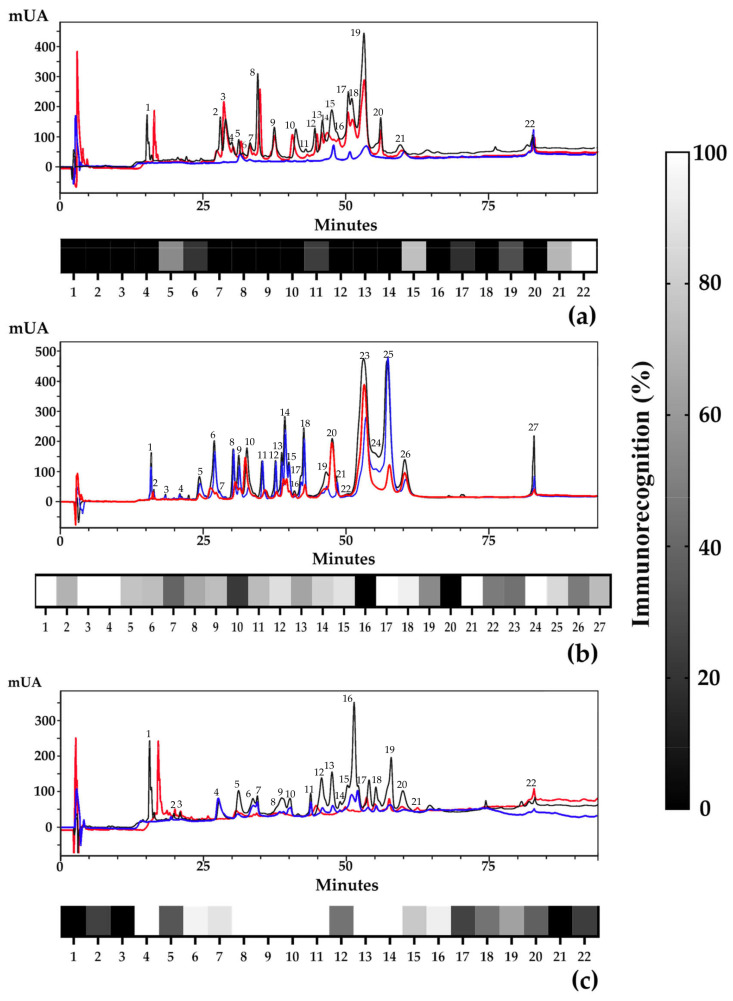
RP-HPLC chromatograms and heat maps of the not retained (red) and retained (blue) fractions from the affinity matrix coupled to anticoral antivenom produced by the Instituto Nacional de Salud. The black solid line shows the chromatogram of the whole venom. The heat maps indicate the immunorecognition of venom fractions by the anticoral antivenom. The immunorecognition percentages for the three venom fractions by RP-HPLC from the immunoaffinity column are based on calculated relative abundances. (**a**) *M. medemi*, (**b**) *M. helleri*, and (**c**) *M. sangilensis*.

Affinity chromatography matrices were coupled with 30 mg of INS antivenom. Overall, 95% of the total protein was not retained (data not shown), which means that the immunospecificity control did not show significant recognition. The fractionation of the coral snake venoms by RP-HPLC showed retention percentages of 15, 62, and 68% for the *M. helleri*, *M. medemi*, and *M. sangilensis* venoms, respectively (Figure 3). Also, RP-HPLC allows for the classification of venom components into three groups (Figure 3). The first group is covered within the first ~38 min when the small hydrophilic molecules appear, such as three-finger toxins (3FTx). Within the following 12 min, the second group, medium-sized molecules with intermediate hydrophobicity, are eluted from the column, such as PLA_2_, C-type lectins, serine proteases, cysteine-rich secretory proteins, and growth factors. The third group of molecules is eluted after minute 50 and includes the most hydrophobic proteins, such as metalloproteases, L-amino acid oxidases, and hyaluronidases, among others, in elapid venoms [13,14,50].

In order to quantify antibody recognition, we calculated the ratio of retained (RET)/unretained fractions (NR) (% NR relative abundance/% RET relative abundance), where values tending toward 0 show the best antibody recognition. The venoms from *M. medemi* and *M. sangilensis* had a mean retention ratio of 0.53, while *M. helleri* had a retention ratio of 5.47. According to the RP-HPLC retention times described above, the best recognition for smaller proteins was observed for *M. sangilensis* (34%), compared to *M. medemi* (22%) and *M. helleri* (12%). Therefore, it is possible to assume that small toxins, like the 3FTxs of *M. sangilensis*, besides being higher in quantity in the venom [23], are better immunogens than those present in the venom of *M. helleri* since their recognition was higher. Fraction 6 from *M. sangilensis* venom was also recognized (95%), and it could be related to 3FTx. It is known that venom neurotoxins of closely related elapid species, such as *M. dumerilii* and *M. mipartitus*, have differences in their chromatographic elution time because of slight amino acid variations in their sequences, suggesting changes in antigenic groups and variability in their recognition by antibodies [51,52].

The average recognition for the second group of molecules in the three venoms was 0.64, indicating a high recognition for medium-sized proteins. The tendency toward antibody recognition was consistent. The venom from *M. sangilensis* showed the highest percentage of recognized proteins (26%); however, the antibody recognition of the other two venoms was around 18%. It is notable that medium-sized proteins, most likely PLA_2_ proteins, which are abundant within the venoms (from 30 to 43%), showed notorious antibody recognition. Yet, fraction 15 from the *M. helleri* venom showed an antibody recognition of 57%.

On the contrary, the best recognition in group three of venom components was observed for *M. helleri* (69%), followed by *M. medemi* (60%) and *M. sangilensis* (40%). These values of antibody recognition may be the consequence of larger-molecular-size proteins that show more immunogenicity due to their exposition to more antigenic regions. The best recognized antibody fractions were fraction 22 from *M. helleri* (76%), fraction 25 from *M. medemi* (84%), and fraction 16 from *M. sangilensis* (94%), which are fractions mainly related to PLA_2_ and proteases [37,50]. It is important to note that the venom of *M. helleri* contains large proteins such as serine proteases, metalloproteases, and L-amino acid oxidases, which represent almost 27% of the *M. helleri* proteome [23] and promote higher antibody recognition. Figure 3 also shows a heat map of the retained fractions eluted from the affinity and RP-HPLC columns, displaying a schematic representation of the presence or absence of fractions according to their size, retention time, and hydrophobicity.

### 2.4. Observed Immunoreactivity against M. sangilensis and M. medemi Is Confirmed by the In Vivo Neutralization Assay

The INS antivenom showed cross-neutralization against the three heterologous venoms (Table 1), exhibiting ED_50_ values even higher than for homologous venoms such as *Micrurus dumerilii* (ED_50_: 0.36 mg of venom/mL antivenom) and *M. surinamensis* (ED_50_: 0.31 mg/mL) but lower compared to *M. mipartitus* (ED_50_: 0.94 mg/mL) and *M. isozonus* (ED_50_: 2.24 mg/mL) venoms [42], given the amount of venom that is neutralized per milliliter of antivenom. Nevertheless, the INS antivenom shows a larger neutralization effect compared to some monovalent and polyvalent antivenoms manufactured in the region; that is, the ED50 for the INS antivenom varies between 3 and 37 times more than the other antivenoms for *Micrurus dumerilii*, *M. mipartitus*, *M. isozonus*, *M. surinamensis*, *M. helleri*, *M. medemi*, and *M. spixii* venoms [42]. For instance, the Instituto Butantan antivenom against *M. corallinus* and *M. frontalis* showed cross-neutralization against *M. helleri* in a proportion of ED_50_ eight times less than that shown with the INS anticoral antivenom [33,35,42].

In this study, the venoms from *M. helleri*, *M. medemi*, and *M. sangilensis* were better neutralized by the INS anticoral antivenom (Table 1) compared to the ICP antivenom from Costa Rica against *M. dumerilii* with an ED_50_ of 0.2 mg/mL (antivenom against *M. nigrocinctus*, *M. carinicaudus*, and *M. fulvius*) [53], but it showed less activity compared to the Bioclon antivenom from Mexico (against *M. nigrocinctus*) [54] and the INBPA antivenom from Argentina (against *M. pyrrhocryptus*) [55] against *M. surinamensis*, with an ED_50_ of 0.03 and 0.4 mg/mL, respectively. However, to date, there are no studies for other anticoral antivenoms produced in Latin America [56] against venoms from *M. helleri*, *M. medemi*, and *M. sangilensis*.

## 3. Conclusions

The INS antivenom showed cross-immunoreactivity against the three elapid venoms, *M. helleri*, *M. medemi*, and *M. sangilensis*, towards most of their components, with *M. sangilensis* being the venom that presented the highest antigen–antibody recognition and neutralization in all the assays. The INS antivenom showed marked predilection for high-molecular-weight proteins and partial recognition for medium/low-molecular-weight proteins in the *M. sangilensis* venom. Although the cross-reactivity of some anticoral antivenoms has been evaluated against other *Micrurus* species, little is known concerning species from Colombia. Therefore, this work shows the cross-reactivity and efficacy of the INS polyvalent antivenom in the clinical management of the envenoming caused by three Colombian *Micrurus*, including different populations of *M. helleri*.

It is important to implement strategies to improve antivenom recognition/neutralization. One of these strategies is to perform neutralization assays with different coral snake species to establish an integrative overview of antivenom efficacy and subsequently reevaluate its recommendation for clinical regions in Colombia.

## 4. Materials and Methods

### 4.1. Venoms and Antivenom

The freeze-dried venom pools of *Micrurus helleri* (Villagarzón—Putumayo, Amazonía region), *M. medemi* (Villavicencio—Meta, Orinoquía region), and *M. sangilensis* (Sutamarchán—Boyacá, Magdalena Medio region) were provided by the Instituto Nacional de Salud de Colombia (INS). An equine-origin IgG, liquid presentation antivenom, manufactured by the INS using *Micrurus dumerilii*, *M. mipartitus*, *M. isozonus*, and *M. surinamensis* venoms, was used as a probe (Batch No. 19AMP02, expiration October/2023).

### 4.2. Protein Quantification

The protein concentration of venoms was determined by the BCA (bicinchoninic acid) method using bovine serum albumin (BSA) as a standard. All the fractions separated in the RP-HPLC were quantified with a nanodrop instrument (Thermo Scientific™ NanoDrop One^©^, Wilmington, DE, USA).

### 4.3. Polyacrylamide Gel Electrophoresis (SDS-PAGE)

The SDS-PAGE gel electrophoresis (15%) was performed according to [57,58]. A Biorad Precision Plus Protein Dual Xtra (2–250 kDa) molecular weight standard was used as a marker. Gels were stained with Coomassie R-250 and analyzed using Bio-Rad’s Image Lab 6.1 software (Bio-Rad Laboratories, Inc., Berkeley, CA, USA, 2020).

### 4.4. Reverse-Phase Liquid Chromatography (RP-HPLC)

RP-HPLC was performed as described in [50]. Briefly, the complete venoms of the affinity column fractions were resuspended in 1 mL of water containing 0.1% trifluoroacetic acid (TFA) (solution A). Subsequently, these samples were subjected to reverse-phase chromatography in a Shimadzu SPD-10A instrument (UV/Vis detector SPD 10) using a Zorbax Eclipse XDB C18 column (4.6 × 250 mm, 5 μm). The samples were eluted with a linear gradient of acetonitrile/TFA 0.1% (solution B) as follows: 0% over 15 min, 0–15% over 15 min, 15–45% over 60 min, 45– 70% for 10 min, 70% for 10 min, 70–100% for 5 min, and sustained at 100% for 5 min. The absorbance was monitored at 215 nm.

### 4.5. Antivenom Assessment

#### 4.5.1. Affinity Chromatography

A second-generation antivenomic technique was used [39]. The venom–antigen coupling was made as described in [59] with some modifications. CNBr-activated sepharose 4B (0.3 g) was packed with 3 mL of prewash buffer (HCl, 1 mM) under stirring for 15 min at room temperature. The matrix was then washed with HCl (1 mM), and a coupling buffer (0.2 M of NaHCO_3_, 0.5 M of NaCl; pH 8.3) was added until pH > 8.5. Subsequently, 30 mg of the INS anticoral antivenom was added in a 1:10 (*v*/*v*) ratio with the resin, previously dialyzed against the coupling buffer, and it was stirred continuously overnight at 4 °C. The supernatant was collected for quantification. The column was then washed with a coupling buffer, blocked with blocking buffer (0.1 M of Tris-HCl; pH 8.0), and left stirring at room temperature for 4 h. To remove the unbound antibodies from the column, six interspersed washes were carried out with a buffer (0.1 M of acetic acid/sodium acetate, 0.5 M of NaCl). The final pH was neutralized with 10 mM of Tris-HCl, pH 8.0. For specificity control, the same procedure was carried out, with IgG from non-immunized horses and *M. helleri* venom used.

A solution of 300 μg of protein from each venom in 400 μL of 10 mM Tris-HCl at pH 8.0 as a buffer was passed thrice through the matrices. The non-retained fraction was eluted with 10 mM of Tris-HCl at pH 8.0; the retained fraction 1 was eluted with 0.1 M of acetic acid (pH 2.4) and neutralized with 1 M of Tris-HCl buffer (pH 8.0); and the retained fraction 2 was eluted with 50 mM of sodium hydroxide. All fractions were neutralized with the same buffer. Each fraction was centrifuged at 13,000 rpm for 2 min. The supernatant was separated and concentrated by ultrafiltration in an Amicon^®^ centrifugal filter device (3 kDa MW cutoff).

#### 4.5.2. Western Blot

Each venom (10 µg of protein) was loaded onto a 15% SDS-PAGE gel under reducing conditions. Subsequently, the protein bands were transferred to a nitrocellulose membrane for 1 h at a constant current of 400 mA in a semi-humid chamber. The membrane was blocked at 4 °C overnight in a blocking buffer (5% skim milk powder, in PBS/0.5% Tween 20; TBST 1X). The membrane was washed three times with TBST 1X and incubated with the primary antibody (INS antivenom) at a dilution of 1:500 in TBST to a final volume of 10 mL under rotating agitation for 1 h at room temperature. Later, the membrane was washed again three times with TBST 1X and incubated with the secondary antibody (KPL Peroxidase-Labeled Antibody to Horse IgG (H+L) Produced in Goat, 0.5 mg), prepared at a dilution of 1:1000 in TBST 1X, and left under rotary agitation for 1 h at room temperature. Finally, the membrane was washed thrice with TBST 1X, and 1 mL of TMB blotting solution was added to reveal [60].

Densitometry gel analysis was performed using GelAnalyzer software v. 23.1.1 (available at www.gelanalyzer.com by Istvan Lazar Jr., PhD and Istvan Lazar Sr., PhD, CSc, accessed on 26 January 2024). The quantitative values of each band were obtained using the volume values of all bands. The corresponding recognition values were calculated by dividing the volume value of each band by the total venom volume.

#### 4.5.3. Enzyme-Linked Immunosorbent Assay—ELISA and EC50 Determination

The samples were prepared in a sensitization buffer (100 mM of carbonate/bicarbonate, pH 9.5) at a concentration of 5 μg/mL. Each sample (100 μL) was seeded in each well in duplicate and incubated at 37 °C for 1 h. The content was discarded, and each well was washed thrice with 200 μL of washing buffer (Tris-HCl: 50 mM, pH 8,0; NaCl: 150 mM). Then, 200 μL of blocking buffer (50 mM of Tris-HCl, pH 8.0, 5 mg/mL of gelatin, 0.02% Tween 20) was added and left at 4 °C overnight. INS antivenom was prepared in 50 mM of Tris-HCl buffer (pH 8.0, 0.5 M of NaCl, 1 mg/mL of gelatin, 0.05% Tween 20) at a concentration of 700 µg/mL. Then, 100 μL was seeded per well, making serial 1:3 dilutions with the vehicle buffer. Each well was previously washed thrice with 200 μL of washing buffer, then left in an incubator at 37 °C for 1 h. All wells were washed thrice with 200 μL of washing buffer, and 100 μL was placed in each well with the secondary antibody preparation (KPL Peroxidase-Labeled Antibody to Horse IgG (H+L) Produced in Goat, 0.5 mg) and dissolved in the vehicle buffer at a 1:4000 dilution. The samples were left in an incubator at 37 °C for 1 h. The plate was washed with washing buffer, and the reaction was developed with ABTS in 70 mM of a citrate–phosphate buffer at pH 4.2 and 0.02 µL of H_2_O_2_. The absorbance was measured with a spectrophotometer at 405 nm 60 min later.

The EC_50_ (half-maximal effective concentration) was calculated as follows: the data obtained from the ELISA procedure were analyzed by nonlinear regression using the sigmoidal dose–response equation of the Prism software (Graph Pad Prism v. 8.3.0, San Diego, CA, USA). Titers were calculated from the midpoint of the curve and correspond to the antivenom dilution for half of the maximal recognition.

#### 4.5.4. In Vivo Neutralization

The neutralization capacity of INS anticoral antivenom was determined using the median effective dose (ED_50_), following WHO guidelines [61,62] and INS standard internal protocols. Solutions containing different concentrations of the antivenom were mixed with three median lethal doses (3LD_50_) per mouse of each species’ venom, as described in the lethality assays shown in [42]. Samples were preincubated at 37 °C for 30 min and then injected intraperitoneally into mice (n = 5 per dose, 500 μL/mice). Five to six different dilutions of the antivenom were tested. The dilution factors ranged between 2.6 and 3.3, attaining concentrations of 0.08 to 32.93 mg/mL. Two negative controls (one with antivenom and one with saline solution, 500 μL/mice) were used. Additionally, a positive control was used (3LD_50_ of venom/mice). The survival time of each animal was recorded for 48 h. The ED_50_ was expressed in milligrams (mg) of venom per milliliter (mL) of antivenom.

### 4.6. Statistical Analysis

A slope-variable nonlinear regression analysis was carried out in order to perform the ELISA assay. All statistical analyses, i.e., determination of mean values, standard deviations, variation coefficients, and 95% confidence intervals, were calculated using Prism 9.0 software (GraphPad, La Jolla, CA, USA). The ED_50_ was determined using the Spearman–Kärber method [63,64,65].

### 4.7. Ethical Statement

All animal procedures were approved by the Ethics Committee for Methodologies and Research of the Instituto Nacional de Salud (CEMIN) through Act 08-2017 of 2 June 2017.

## Figures and Tables

**Table 1:** Neutralization effectiveness of the Instituto Nacional de Salud anticoral antivenom against *M. helleri*, *M. medemi*, and *M. sangilensis*.

Venom	Median Effective Dose (ED_50_) *
*Micrurus helleri*	0.58 (0.4–0.84) **
*Micrurus medemi*	0.68 (0.44–1.06) **
*Micrurus sangilensis*	0.75 (0.53–1.07) **

* ED_50_ values are expressed in mg of venom/mL antivenom; ** in brackets, the 95% confidence limits.

## Data Availability

Data are contained within the article.
